# Spontaneous Brain Activity in Type 2 Diabetics Revealed by Amplitude of Low-Frequency Fluctuations and Its Association with Diabetic Vascular Disease: A Resting-State fMRI Study

**DOI:** 10.1371/journal.pone.0108883

**Published:** 2014-10-01

**Authors:** Chun-Xia Wang, Kai-Liang Fu, Huai-Jun Liu, Fei Xing, Song-Yun Zhang

**Affiliations:** 1 Department of Medical Imaging, the Second Hospital of Hebei Medical University, Shijiazhuang, Hebei, China; 2 Department of Endocrinology, the Second Hospital of Hebei Medical University, Shijiazhuang, Hebei, China; Cuban Neuroscience Center, Cuba

## Abstract

**Purpose:**

To investigate correlations between altered spontaneous brain activity, diabetic vascular disease, and cognitive function for patients with type 2 diabetes mellitus (T2DM) using resting-state functional magnetic resonance imaging (rs-fMRI).

**Methods:**

Rs-fMRI was performed for T2DM patients (n = 26) and age-, gender-, and education-matched non-diabetic control subjects (n = 26). Amplitude of low frequency fluctuations (ALFF) were computed from fMRI signals to measure spontaneous neuronal activity. Differences in the ALFF patterns between patients and controls, as well as their correlations with clinical variables, were evaluated.

**Results:**

Compared with healthy controls, T2DM patients exhibited significantly decreased ALFF values mainly in the frontal and parietal lobes, the bilateral thalumi, the posterior lobe of the cerebellum, and increased ALFF values mainly in the visual cortices. Furthermore, lower ALFF values in the left subcallosal gyrus correlated with lower ankle-brachial index values (*r* = 0.481, *p* = 0.020), while lower ALFF values in the bilateral medial prefrontal gyri correlated with higher urinary albumin-creatinine ratio (*r* = −0.418, *p* = 0.047). In addition, most of the regions with increased ALFF values in the visual cortices were found to negatively correlate with MoCA scores.

**Conclusions:**

These results confirm that ALFF are altered in many brain regions in T2DM patients, and this is associated with the presence of diabetic vascular disease and poor cognitive performance. These findings may provide additional insight into the neurophysiological mechanisms that mediate T2DM-related cognitive dysfunction, and may also serve as a reference for future research.

## Introduction

There has been growing concern regarding the brain damage associated with diabetes mellitus, especially cognitive dysfunction associated with type 2 diabetes mellitus (T2DM) [1]–[3]. It has previously been demonstrated that patients with diabetes have an increased risk of dementia. For example, in a recent comprehensive meta-analysis of more than 40,000 subjects, aggregate relative risks for Alzheimer disease and vascular dementia (VaD) were found to be 1.5 and 2.5, respectively [4]. T2DM has also been associated with declines in multiple cognitive domains, including psychomotor speed, learning, and memory [1], [5]. However, the factors responsible for these effects are complex.

It has been proposed that vascular components may play a key role in the pathogenesis of cognitive dysfunction in diabetics [1], [3], [6] since vascular components have a central role in mediating VaD and also affect the pathogenesis of Alzheimer disease [7]. It is well-established that T2DM patients are predisposed to macro- and microvascular complications throughout the body, and this can include cerebral small vessel disease (SVD). SVD manifests on brain MRI as lacunar infarcts and white matter lesions (WMLs), and is associated with cognitive impairment [8], [9]. Peripheral artery disease (PAD) is a macrovascular disease that commonly accompanies T2DM. Currently, the ankle-brachial index (ABI) is the most effective and accurate noninvasive method for evaluating PAD [10], and PAD has been associated with cognitive decline and dementia in both diabetic and non-diabetic populations [11]–[13]. Albuminuria is an early marker of diabetic nephropathy and can reflect the extent of diabetic microvascular disease. The urinary albumin-to-creatinine ratio (UACR) is commonly used to evaluate albuminuria, and in some cases, has been an indicator of cognitive decline [14]. For example, in elderly T2DM patients, albuminuria was found to be associated with frontal lobe dysfunction independent of cerebral SVD [15]. Despite these observations, however, the exact neuropatho-physiological mechanisms that mediate cognitive impairment related to T2DM and its vascular complications have not been fully elucidated.

Using structural MRI, atrophy has been detected in certain brain regions of patients with T2DM. The brain regions that are most frequently affected include the medial temporal lobe (hippocampus) and the prefrontal lobe [16]–[19]. Moreover, the volume of these regions has been found to be negatively influenced by poor glycemic control [16]–[18]. In work by Last and coworkers, cerebral blood flow in T2DM patients was evaluated using MRI continuous arterial spin labeling, and hypoperfusion of the frontal and temporal regions was observed [20]. However, a correlation analysis between those regions and cognitive function in T2DM patients versus controls was either absent or not significant. When diffusion tensor imaging was used, a correlation between microstructural abnormalities of white matter and cognitive function were observed in T2DM patients [21]. However, neuron activity could not be monitored using this method.

Functional neuroimaging represents a useful technique for exploring the neurophysiological mechanism(s) underlying various clinical disorders. Correspondingly, functional connectivity has been used to detect brain abnormalities in T2DM patients [22], [23]. Moreover, reduced functional connectivity was found in the default mode network [22], as well as between the hippocampus and more widespread regions of the brain [23]. The amplitude of low frequency fluctuations (ALFF) for blood oxygen level-dependent signals is another analysis algorithm for resting-state functional MRI (rs-fMRI), and is defined as the mean square root of the power spectrum density over the low frequency band (usually 0.01–0.08 Hz) [24]. ALFF has proven to reflect intrinsic brain activity and to be of physiological significance [25], [26]. Unlike functional connectivity, which measures the synchronicity of neuron activity signals among remote regions of the brain, ALFF can provide information regarding regional spontaneous activity. Correspondingly, ALFF has been used to map differences in spontaneous brain activity between patients with various diseases and healthy controls [27], [28]. However, to date, only a few studies [29] have applied ALFF to T2DM-related cognitive dysfunctions, and the relationship between altered ALFF and diabetic vascular disease has not been evaluated.

Based on the results of previous studies that T2DM-related cognitive dysfunction is closely associated with diabetic vascular damage [1], [3], [6] and ALFF sensitivity can detect the spontaneous activity of neurons [25], [26], the aim of the current study was to compare ALFF patterns between T2DM patients and healthy controls, and to determine whether alterations in these patterns correlate with diabetic variables, particularly vascular variables such as ABI and UACR, and cognitive dysfunction. It is anticipated that these results will provide valuable insight into the neurophysiological mechanisms that mediate cognitive decline in diabetics. Furthermore, this information may also facilitate the early diagnosis and timely intervention for cognitive dysfunction due to diabetes.

## Materials and Methods

### Participants

This study was approved by the review board of the Second Hospital of Hebei Medical University, and written informed consent was obtained from all participants prior to their participation.

The study cohort consisted of 26 T2DM patients and 26 age-matched healthy controls. The former were outpatients recruited from the Endocrinology Department of the Second Hospital of Hebei Medical University, China. Patient inclusion criteria included: a patient age between 35 and 70 y, disease duration of at least one year, and right-handedness. All patients met the World Health Organization 1999 criteria [30] for T2DM. In addition, 12 patients were being treated with metformin and insulin, while 14 patients were being treated with metformin and a glucagon-like peptide-1 analogue. Patients treated with insulin secreting drugs, such as sulfonylureas and nateglinide, were excluded from this study in order to eliminate the potential influence of these drugs on brain function [31], [32]. Furthermore, none of the patients examined had a history of severe hypoglycemic episodes as defined by seizures, loss of consciousness, or the need for another person to help treat the symptoms of low blood glucose [33]. In contrast, healthy controls were recruited from the community via advertisements, and were matched according to age, gender, body mass index (BMI), smoking status, hypertension, dyslipidemia, and education. Control individuals with a fasting glucose >6.1 mmol/l, or a postprandial glucose >7.8 mmol/l, were excluded. Exclusion criteria for all participants included: a history of known stroke, alcoholism, head trauma, Parkinson's disease, any kind of neurological or psychiatric illness (excluded by clinical assessment and case history), major medical illness (e.g. cancer, anemia and chronic kidney disease), autoimmune disease, other kinds of endocrine disease, and severe visual or hearing loss. Any contraindications to imaging, such as coronary stenting, pacemaker, pregnancy, and claustrophobia, were also factors for exclusion.

### Clinical data and neuropsychological tests

Clinical data were collected, including weight, height, BMI  =  (weight in kg)/(height in m) ^2^, education years, handedness, smoking status, alcoholic intake, hypertension and antihypertensive agent use, dyslipidemia and anti-dyslipidemic agents. Subjects with blood pressure (BP) >140/90 mm Hg at three different time points, or those taking antihypertensive drugs, were defined as hypertensive. Blood samples were obtained by venepuncture following overnight fasting at 8 A.M., and then following a regular breakfast at 10 A.M., to assess levels of fasting blood glucose, triglycerides, total cholesterol, low density lipoprotein cholesterol, high density lipoprotein cholesterol, and postprandial glucose. Dyslipidemia was detected when triglycerides, total cholesterol, low density lipoprotein cholesterol, or high density lipoprotein cholesterol exceeded the corresponding reference ranges. For T2DM patients, levels of fasting serum C-peptide and glycated hemoglobin (HbA1c) were also assayed. In addition, fresh midstream urine samples were collected in the morning to test urine albumin and creatinine concentrations. The resulting UACR was calculated in mg/mmol creatinine.

To evaluate the neuropsychological status of all of the subjects, cognitive tests were performed. Participants completed the Mini Mental State Exam (MMSE), the Montreal Cognitive Assessment (MoCA), and the Clock Drawing Test (CDT). The selection of these tests was based on their use in previous studies that had reported cognitive dysfunction in T2DM patients [23], [34], and also their straightforward application in clinical practice. The MMSE measures five cognitive domains (e.g., orientation, registration, attention and calculation, recall, and language and praxis), while the MoCA measures seven cognitive domains, including domains that are not measured by the MMSE, such as executive function and abstraction. Both the MMSE and the MoCA exams assess cognition using a scale of 0–30 points, while the CDT uses a scale of 0–4 points. Approximately 30 min were needed for each subject to complete all of the tests in a fixed order. A trained neuropsychiatrist facilitated this process and was not informed of each subjects' identity as a control or patient. Also, none of the participants exhibited audiovisual or motor coordination deficits that would affect the neuropsychological tests.

To obtain ABI values, a portable 8 MHz Doppler device (Huntleigh MD2 Cardiff, CF24 5HN, UK) was attached to a standard sphygmomanometer to simultaneously measure bilateral brachial and ankle systolic BPs. Systolic BP was obtained at the ankle (i.e., the posterior tibial and pedious arteries) and arms (i.e., the brachial artery) on both sides. ABI was calculated as the systolic BP ratio for the ankle and the ipsilateral arm, and was determined for each leg. The lowest ABI value for each individual was used to identify PAD according to the commonly applied criteria of an ABI value <0.9 [12].

### MRI procedures

All subjects were scanned using a 3.0T MRI scanner (Phillips Achieva 3.0T TX) with an 8-channel head coil. Subjects were supine with their head fixed with foam pads to minimize head motion. Earplugs were used to reduce scanner noise. The subjects were instructed to lie quietly with their eyes closed, not to fall asleep, not to think of anything in particular, and to avoid head motion during the MRI.

Functional images were collected axially using an echo-planar imaging (EPI) sequence as follows: repetition time (TR)  = 2000 ms; echo time (TE)  = 30 ms; slices  = 33; thickness  = 5 mm; gap  = 0 mm; field-of-view (FOV)  = 192×192 mm^2^; acquisition matrix  = 64×64; and flip angle  = 90°. The scan time was 7 min. High-resolution three-dimensional T1-weighted sagittal images covering the entire brain were acquired using a turbo-field echo sequence with the following parameters: TR  = 7.7 ms; TE  = 3.8 ms; slices  = 180; thickness  = 1 mm; gap  = 0 mm; flip angle  = 8°; acquisition matrix  = 252×227; and FOV  = 250×250 mm^2^. The scan time was 2 min and 58 s.

The conventional imaging protocol consisted of a T2-weighted fast spin echo (TR/TE  = 2000/80 ms). Fluid-attenuated inversion-recovery (FLAIR) (TR/TE  = 9000/140 ms) sequences in the axial plane with a slice thickness of 5 mm and a 2-mm interslice gap were also acquired. Subjects with large vessel infarcts (n = 2) and severe WMLs (n = 5, as defined by Umenura et al. [35]) on conventional images were excluded based on their potential to influence ALFF values and cognitive function.

### Image preprocessing

Data preprocessing was conducted using the Data Processing Assistant for Resting-State fMRI (DPARSF) program [36] which are based on statistical parametric mapping software (SPM8, http://www.fil.ion.ucl.ac.uk/spm). The first ten volumes were discarded since they included the establishment of signal equilibrium and the adaptation of subjects to the imaging circumstances. The remaining 190 consecutive volumes were analyzed. The following steps were subsequently made: slice-timing adjustment, realignment for head-motion correction, spatial normalization to the Montreal Neurological Institute (MNI) space using a unified segmentation algorithm (resampling voxel size  = 3×3×3 mm^3^) and smoothing using an isotropic Gaussian kernel (FWHM  = 4 mm), and detrend and filtering (0.01–0.08 Hz). Two T2DM patients with head movement exceeding 2.0 mm of maximum translation in any of the x, y, and z directions, or 2.0 degrees of maximum rotation about the three axes, were excluded to minimize movement artifacts. ALFF values were calculated as previously described [37], [38]. Briefly, time courses were first converted to the frequency domain using a Fast Fourier Transform. The square root of the power spectrum was computed and the average was squared across 0.01–0.08 Hz at each voxel. This averaged square root represents the ALFF value [37]. For standardization purposes, the ALFF value for each voxel was divided by the global mean ALFF value. ALFF computations and further analyses were performed within a grey matter (GM) mask that corresponded to the automated anatomical labeling atlas [39].

### Structural voxel-based morphometry (VBM) segmentation

Structural MRI studies have suggested that T2DM patients exhibit GM loss in many regions of the brain [19]. Thus, GM loss may affect imaging of function. To address this issue, high-resolution three dimensional T1 images were subjected to VBM analysis using DPARSF software. Briefly, the T1 images were segmented into GM, white matter, and cerebrospinal fluid using unified segmentation, and then these segments were normalized to MNI space. The resulting GM maps were modulated and smoothed using a 4 mm Gaussian kernel. The resulting images were subsequently analyzed as a covariate (and were not analyzed between the two groups).

### Classical markers of SVD

A quantitative assessment of WMLs was performed using FLAIR images and T2-weighted images. Age-related white matter changes were scored [40] by two of the authors (K-L. Fu, F. Xing) who were blinded to the clinical data and group allocations. Five different regions in each of the right and left hemispheres were rated separately, then were added to represent the global WML burden. Lacunar infarcts were also identified. Consensus was obtained regarding disagreements.

### Statistical analysis

Demographic variables, clinical variables, and cognitive performance scores were compared using SPSS software (version 17.0; SPSS, Inc., Chicago, IL, USA). Distribution of quantitative variables was evaluated using the Shapiro-Walk test. Data that did not exhibit a normal distribution were transformed using a base-10 logarithm for further analysis. Differences in demographic, clinical, and cognitive data between patients and healthy controls were analyzed using either a group t-test for normally distributed data or a Wilcoxon two-sample test for data that did not achieve normal distribution after logarithm transformation and a χ^2^-test for proportions. Cognitive data analyses were adjusted for age, gender, and education levels. All tests were conducted using a two-sided α-level of 0.05. Using the homeostasis model assessment version 2 (HOMA2) available from the Diabetes Trials Unit website (http://www.dtu.ox.ac.uk) [41], [42], insulin sensitivity (HOMA2-S%) was assessed based on fasting plasma glucose (FPG) and fasting C-peptide levels.

#### Voxel-wise ALFF analysis

To perform voxel-based ALFF analyses, a rs-fMRI data analysis toolkit was used (REST, http://www.restfmri.net [24]). First, one-sample t-tests were performed for both T2DM and healthy control groups to evaluate within-group whole brain ALFF patterns. Statistical significance was set at voxel-wise *p*<0.01 and a cluster size of 21 voxels which corresponded to a whole-brain corrected *p*<0.01. This correction was determined by Monte Carlo simulations [43] and was performed using the REST AlphaSim program with the following parameters: single voxel *p* = 0.01, FWHM  = 4 mm, and 1000 iterations, within a customized, automated anatomical labeling GM mask [39]. Second, two-sample t-tests were employed to investigate between-group differences of ALFF values (within the GM mask). Age, BMI, GM volume, and WMLs were included as nuisance covariates to control for the possible influence of these factors on the results. The statistical threshold was set at voxel-wise *p*<0.05 and a cluster size of 46 voxels which corresponded to a whole-brain corrected *p*<0.05 (AlphaSim correction with parameters: single voxel *p* = 0.05, FWHM  = 4 mm, and 1000 iterations). Third, to investigate which brain regions correlated with MoCA scores, linear correlations were calculated between ALFF values and MoCA scores (within the GM mask), adjusted for patient age, education, WMLs, and GM volume. The threshold was set at voxel-wise *p*<0.05 and a cluster size of 46 voxels which corresponded to a whole-brain corrected *p*<0.05 (AlphaSim correction with parameters: single voxel *p* = 0.05, FWHM  = 4 mm, and 1000 iterations).

To investigate the relationship between altered ALFF values in T2DM patients, various diabetic variables (e.g., diabetes duration, HbA1c, HOMA2-S%, ABI, and UACR), and MoCA scores, the mean ALFF values for each of the survived clusters were extracted separately. Partial correlations between the extracted ALFF values, diabetic variables, and MoCA scores were then analyzed using SPSS software. Correlations were controlled for patient age, gender, and education. A *p*-value less than 0.05 was considered statistically significant.

## Results

### Clinical and neuropsychological data

Table 1 reveals the demographic, clinical, and neuropsychological characteristics of the T2DM patients and healthy controls included in this study. There were no significant differences observed between the two groups in terms of patient age, gender, education level, BMI, hypertension, smoking status, and dyslipidemia conditions. Moreover, the results of the MMSE test did not significantly differ between the two groups (*p*>0.05). However, T2DM patients did exhibit higher fasting glucose and postprandial glucose levels (both *p*<0.001), as well as lower MoCA and CDT scores, after correcting for patient age and education (*p* = 0.007 and *p* = 0.002, respectively).

**Table 1:** Demographic, clinical, and cognitive characteristics of T2DM patients and healthy control subjects.*

Characteristics	T2DM patients	Control subjects	*p*-value
	(n = 26)	(n = 26)	
Age (y)	54.7±10.4	54.9±9.8	0.946
Gender (Male/Female)	17/9	17/9	-
Education (y)	11.2±3.8	10.7±3.2	0.586
BMI (kg/m^2^)	25.9±3.3	25.7±3.3	0.851
Hypertensive, n (%)	10 (38.5)	11 (42.3)	0.778
Current smokers, n (%)	10 (38.5)	9 (34.6)	0.773
Dyslipidemia, n (%)	9 (34.6)	10 (38.5)	0.773
Fasting glucose (mmol/L)	9.3±2.0	5.2±0.4	<0.0001**
Postprandial glucose (mmol/L)	13.8±4.4	6.6±0.8	<0.0001**
Diabetes duration (y), median (range)	7 (1–26)	-	-
HbA1c (%)	8.3±1.4	-	-
Fasting C-peptide (nmol/L)	0.53±0.18	-	-
HOMA2-S%	81.6±39.4	-	-
ABI	1.02±0.16	-	-
UACR, median (range)	1.0 (0.2–16)	-	-
MMSE	27.8±2.5	28.3±1.3	0.137
MoCA, median (range)	24.0 (17–29)	26.5 (19–30)	0.007**
CDT, median (range)	2.5 (1–4)	4 (1–4)	0.002**
Classical MRI markers of SVD			
Large vessel infarcts	None	None	-
Lacunar infarcts, n (%)	2 (7.7)	2 (7.7)	-
WMLs, median (range)	4 (0–10)	4 (0–10)	0.500

*Data are presented as the mean ± SD unless otherwise noted; ** *p*<0.05.

T2DM, type 2 diabetes mellitus; BMI, body mass index; HbA1c, glycated hemoglobin; HOMA2-S%, homeostasis model assessment for insulin sensitivity; ABI, ankle-brachial index; UACR, urine albumin creatinine ratio; MMSE, Mini Mental State Exam; MoCA, Montreal Cognitive Assessment; CDT, clock drawing test; MRI, magnetic resonance imaging; SVD, small vessel disease; WMLs, white matter lesions (these were assessed in both hemispheres using the Wahlund age-related white matter changes scale [40]).

### Diabetic vascular disease

Neither the T2DM patients nor the controls exhibited large vessel infarcts. The global WML burden score for both groups ranged from 0–10 (median score, 4). These values were relatively mild considering that 30 was the highest score available. Furthermore, lacunar infarcts were only detected in two T2DM patients and two controls. Therefore, there was no significant difference in WMLs and lacunar infarcts for the two groups (both *p*>0.05).

Using the commonly applied criteria of an ABI value <0.90 to define PAD [12], the incidence of PAD for the T2DM patients was found to be 30.8% (8/26). However, an ABI value <1.0 may also indicate the presence of a subclinical disease, since the sensitivity and specificity associated with this ABI value for screening coronary heart disease was found to be 41% and 73%, respectively [13]. Thus, if an ABI value <1.0 was used as the criteria for defining PAD, the incidence of PAD in the current study would be 46.2% (12/26). Using the criteria recommended by the American National Kidney Foundation to define albuminuria [44], macroalbuminuria (i.e., UACR >33.9 mg/mmol) was not detected in any of the T2DM patients. However, the incidence of microalbuminuria (i.e. UACR >1.92 g/mol for males and >2.83 g/mol for females) in T2DM patients was 19.2% (5/26).

### ALFF data

**Figure 1:**
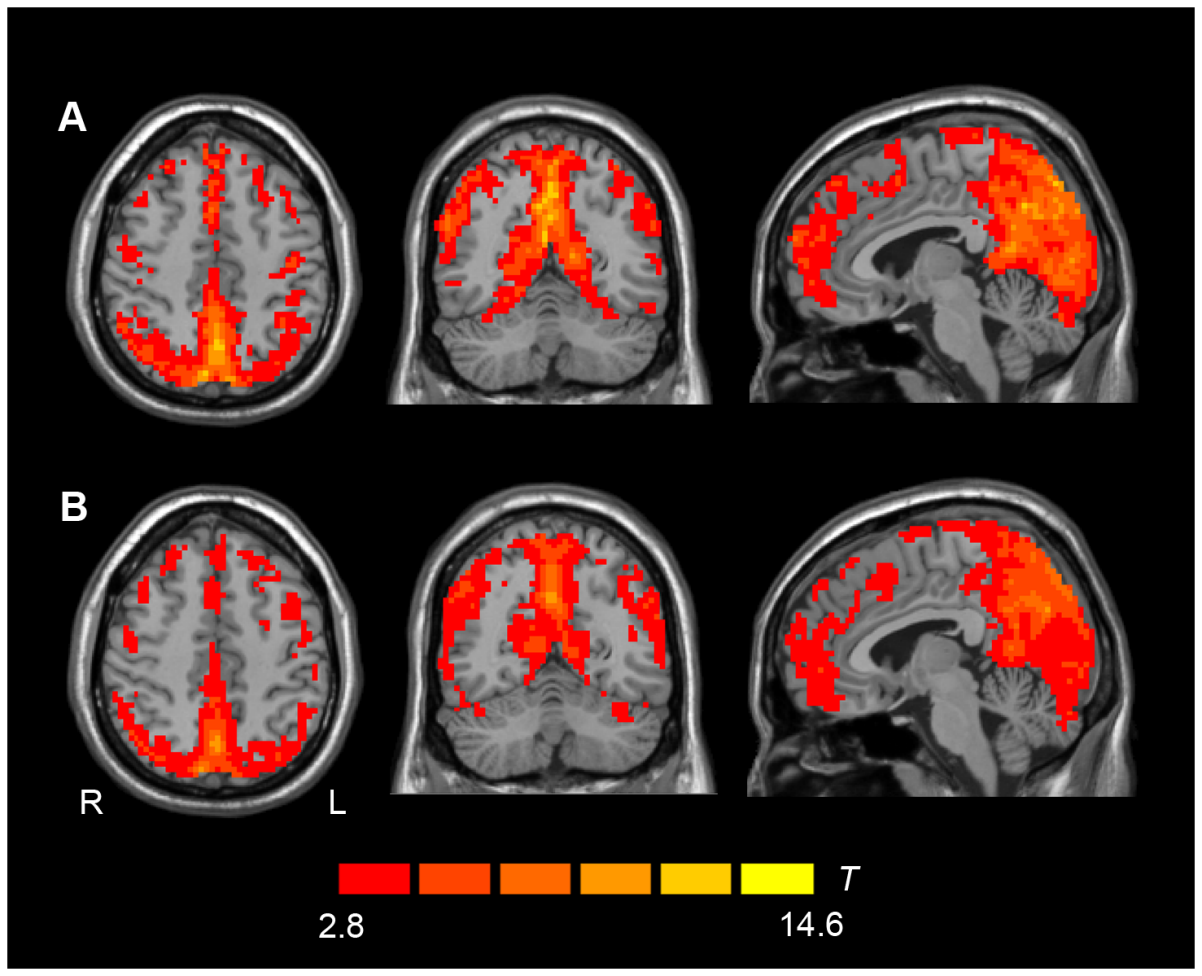
Activation ALFF maps using one sample t-test. ALFF patterns detected in T2DM patients (A) and healthy controls (B). *P*<0.01, AlphaSim correction was applied using a minimum cluster size of 21 voxels. The underlying structure image is *Ch2* image.

Using a one-sample t-test, similar ALFF activation patterns were observed for T2DM patients and controls. Moreover, the regions with significantly higher ALFF values compared to the global mean were mainly within the DMN for both groups, and this included the posterior cingulated cortex, the adjacent precuneus, the medial prefrontal cortex (MPFC), and the inferior parietal lobule (IPL). The cerebellum posterior lobe and cuneus also exhibited higher spontaneous activity (Figure 1).

**Figure 2:**
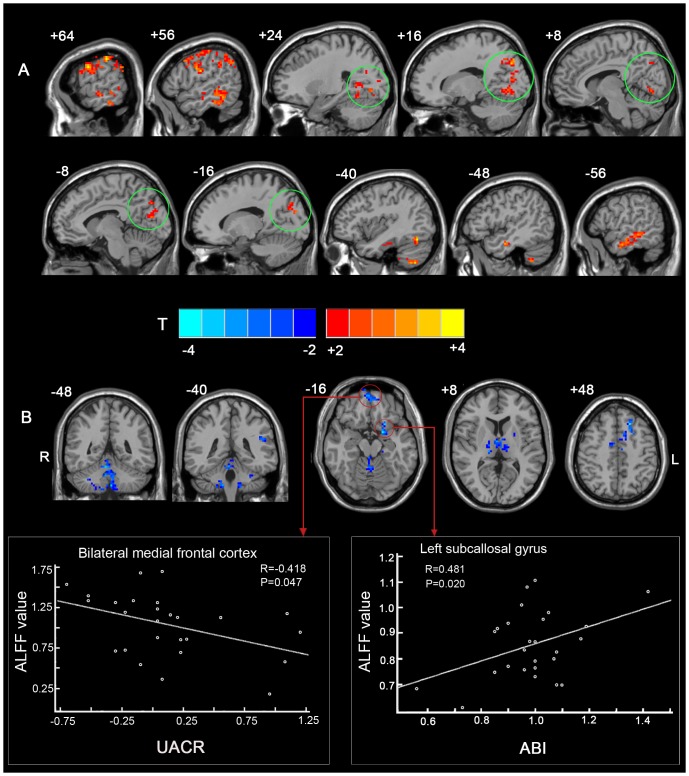
Altered ALFF in T2DM patients compared to healthy controls. (A) Regions of increased ALFF in T2DM patients. The visual network is indicated with green circles. (B) Regions of decreased ALFF in T2DM patients. The scatter-plot maps below show the correlations calculated between ALFF values for the clusters indicated with red arrows and indices of diabetic vascular disease (e.g., UACR and ABI). Note that the UACRs have been logarithm transformed. *P*<0.05, AlphaSim correction was applied using a minimum cluster size of 46 voxels. The underlying structure image is *Ch2* image.

Using a two-sample t-test analysis, T2DM patients showed a significant decrease in ALFF values for the bilateral MPFC, the left subcallosal gyrus, the left IPL, the left supplementary motor area, the bilateral thalamus, the bilateral cerebellum region 9, and the vermis. Furthermore, increased ALFF values were present in widespread visual network (including the parietal-temporal lobe) and the left cerebellum region 7b (Figure 2, Table 2).

**Table 2:** Brain regions that showed significant differences in ALFF values for the T2DM group compared with the healthy control group.

Brain regions	BA	x, y, z (MNI)	*T*-value	Voxels
**(I) Increased ALFF**				
L cerebellum-7b	NA	−45, −51, −51	3.7409	57
R middle and inferior temporal gyrus	21/20	60, −36, −9	3.3025	219
L inferior and middle temporal gyrus	20/21	−51, −9, −24	3.9625	225
L fusiform gyrus	19/37	−39, −66, −18	3.7928	57
R lingual and fusiform gyrus	18/19/37	27, −75, −6	3.6172	169
R middle occipital gyrus	18/19	30, −81, 9	3.4724	56
R precuneus and cuneus	7/18/19	12, −69, 36	3.6738	97
L cuneus and precuneus	18	−18, −72, 24	3.5743	82
R pre- and postcentral gyri and IPL	2/3/4/6/40	69, −21, 30	4.1422	465
**(II) Decreased ALFF**				
B medial prefrontal gyri	11	−6, 60, −15	−3.2125	46
L subcallosal gyrus	25	−18, 3, −15	−3.6999	55
L inferior parietal lobule	40	−57, −33, 27	−3.3008	49
L superior frontal gyrus	6	−18, 27, 48	−3.8333	127
L supplementary motor area	6	−6, −9, 54	−2.9262	64
B cerebellum_9	NA	12, −42, −57	−3.5920	74
Cerebellar vermis	NA	6, −51, −21	−3.9707	227
B thalumi	NA	15, −15, 12	−3.3197	79

A corrected threshold of *p*<0.05 was applied according to Monte Carlo simulations.

BA, Brodmann's area; MNI, Montreal Neurological Institute; L, left; R, right; NA, not applicable; B, bilateral.

### Correlation between extracted ALFF values from survived clusters and clinical variables for T2DM patients

There were 17 survived clusters which exhibited significantly altered ALFF values in the T2DM patients compared with the controls. Partial correlation analyses between the ALFF value of each cluster and the clinical variables evaluated revealed that the ALFF values for two of the clusters correlated with ABI and UACR values, respectively (Figure 2, Table 3), while the ALFF values of six other clusters correlated with MoCA scores (Table 3). Specifically, the ALFF values in the left subcallosal gyrus correlated positively with ABI (*r* = 0.481, *p* = 0.020), and the ALFF values in the bilateral MPFC correlated negatively with UACR (*r* = −0.418, *p* = 0.047) (Figure 2). The regions with ALFF values that negatively correlated with the MoCA scores were located in the bilateral occipital lobe (e.g., the bilateral cuneus and precuneus, the right lingual and fusiform gyri, and the right middle occipital gyrus) and the bilateral cerebellum region 9. Conversely, the regions with ALFF values that positively correlated with the MoCA scores were located in the left cerebellum region 7b. However, no correlations were detected between any of the survived cluster and diabetes duration, HbA1c, or HOMA2-S% values (Table 4).

**Table 3:** Partial correlations between the ALFF values of the survived clusters, the vascular variables evaluated, and the MoCA scores for the T2DM patients.

Cluster ALFF values	ABI		UACR		MoCA score	
	R-value	P-value	R-value	P-value	R-value	P-value
**(I) Increased ALFF**						
L cerebellum-7b	0.108	0.625	0.027	0.901	0.461	0.027*
R middle and inferior temporal gyrus	0.305	0.157	0.083	0.707	0.287	0.184
L inferior and middle temporal gyrus	0.367	0.085	0.052	0.815	0.309	0.151
L fusiform gyrus	−0.326	0.129	−0.051	0.817	−0.137	0.533
R lingual and fusiform gyrus	−0.124	0.573	0.093	0.674	−0.559	0.006*
R middle occipital gyrus	−0.083	0.708	0.148	0.500	−0.484	0.019*
R precuneus and cuneus	−0.125	0.570	0.000	0.998	−0.415	0.049*
L cuneus and precuneus	−0.209	0.339	−0.032	0.886	−0.458	0.028*
R pre- and postcentral gyri and IPL	0.349	0.102	0.105	0.635	0.005	0.981
**(II) Decreased ALFF**						
B medial prefrontal gyri	−0.065	0.768	−0.418	0.047*	0.114	0.604
L subcallosal gyrus	0.481	0.020*	−0.095	0.665	0.068	0.759
L inferior parietal lobule	0.090	0.683	0.088	0.689	0.252	0.246
L superior frontal gyrus	0.192	0.379	0.233	0.284	0.154	0.482
L supplementary motor area	0.171	0.435	0.215	0.324	0.111	0.614
B cerebellum_9	0.032	0.883	−0.298	0.167	−0.478	0.021*
Cerebellar vermis	0.313	0.145	−0.153	0.485	−0.083	0.707
B thalumi	0.203	0.354	−0.087	0.692	−0.011	0.962

These analyses were controlled for patient age, gender, and education level, * *p<*0.05.

ABI, ankle-brachial index; UACR, urinary albumin creatinine ratio; MoCA, Montreal Cognitive Assessment; L, left; R, right; B, bilateral.

**Table 4:** Partial correlations between the ALFF values of the survived clusters and the diabetic clinical variables examined for the T2DM patients.

Cluster ALFF values	DM duration		HbA1c		HOMA2-S%	
	R-value	P-value	R-value	P-value	R-value	P-value
**(I) Increased ALFF**						
L cerebellum-7b	0.119	0.587	0.159	0.468	0.038	0.863
R middle and inferior temporal gyrus	−0.159	0.469	0.273	0.207	−0.174	0.428
L inferior and middle temporal gyrus	−0.108	0.625	0.224	0.304	−0.367	0.085
L fusiform gyrus	0.337	0.116	−0.054	0.808	0.347	0.105
R lingual and fusiform gyrus	0.168	0.442	−0.147	0.505	0.038	0.864
R middle occipital gyrus	0.253	0.245	−0.057	0.796	0.010	0.965
R precuneus and cuneus	0.387	0.068	−0.195	0.374	0.000	0.998
L cuneus and precuneus	0.135	0.539	−0.122	0.579	0.077	0.762
R pre- and postcentral gyri and IPL	0.019	0.932	0.286	0.186	−0.082	0.709
**(II) Decreased ALFF**						
B medial prefrontal gyri	0.153	0.487	−0.132	0.547	0.413	0.051
L subcallosal gyrus	−0.120	0.586	0.072	0.744	−0.361	0.091
L inferior parietal lobule	−0.052	0.812	0.049	0.823	−0.316	0.142
L superior frontal gyrus	−0.035	0.874	−0.320	0.136	−0.089	0.686
L supplementary motor area	−0.141	0.520	−0.115	0.603	0.019	0.931
B cerebellum_9	−0.070	0.751	−0.44	0.841	−0.111	0.614
Cerebellar vermis	0.050	0.819	0.040	0.857	−0.238	0.274
B thalumi	−0.028	0.899	0.242	0.267	−0.326	0.128

These analyses were controlled for patient age, gender, and education level. No significant correlations were observed in these analyses.

DM, diabetes mellitus; HbA1c, glycated hemoglobin; HOMA2-S%, homeostasis model assessment for insulin sensitivity; L, left; R, right; B, bilateral.

### Correlation between full-brain ALFF values and MoCA scores in a voxel-wise manner

**Figure 3:**
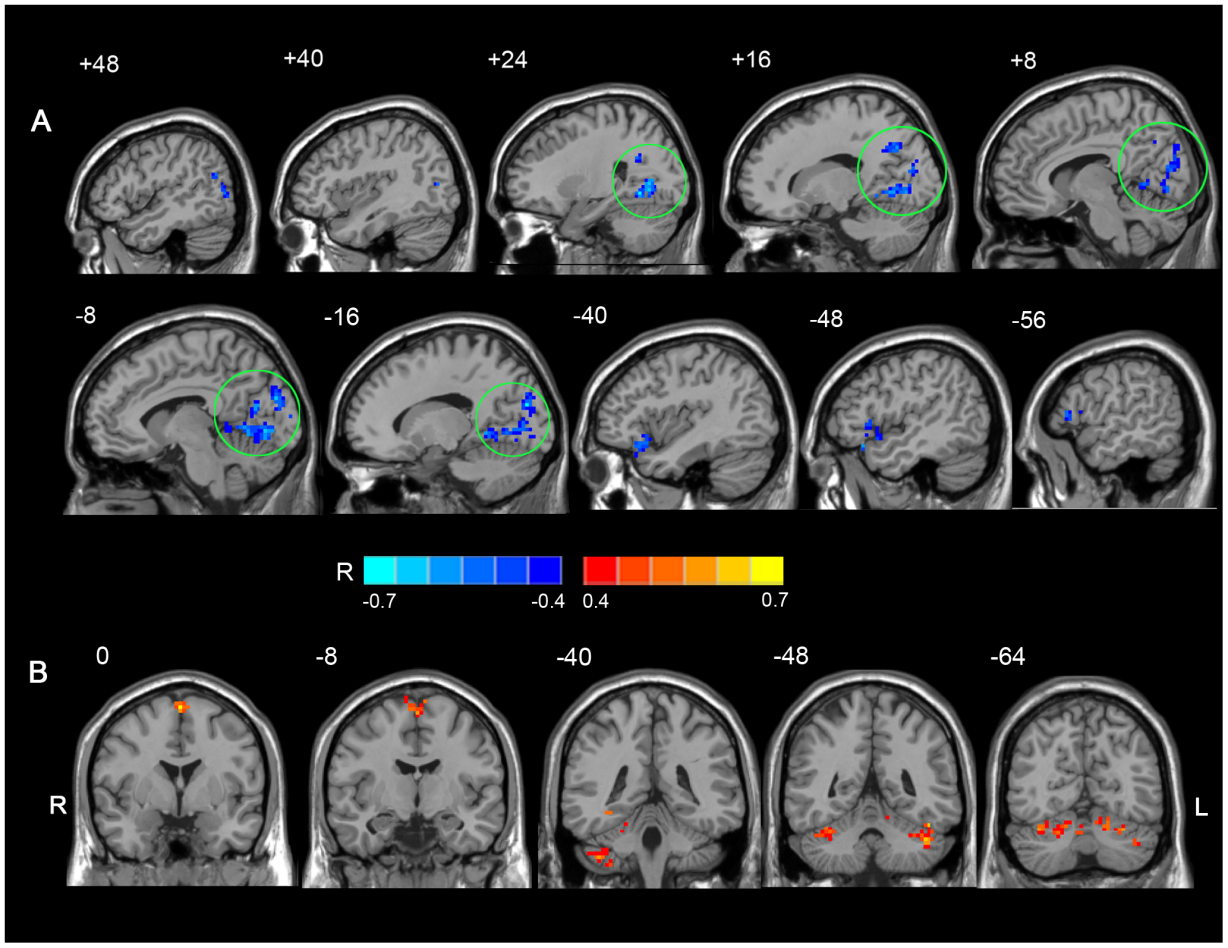
Regions of ALFF values that correlated with MoCA scores in T2DM patients. (A) Regions that negatively-correlated with MoCA scores in T2DM patients. Note that the visual cortex is indicated with green circles and these regions are highly consistent with the data shown in Figure 1A. (B) Regions that positively-correlated with MoCA scores in T2DM patients. *P*<0.05, AlphaSim correction was applied using a minimum cluster size of 46 voxels. The underlying structure image is *Ch2* image.

A correlation analysis between mean ALFF values and MoCA scores in a voxel-wise manner was performed for T2DM patients. A positive correlation was identified for ALFF values mainly in the bilateral supplementary motor areas and the bilateral cerebellum. In contrast, the left inferior frontal gyrus and the bilateral occipital and temporal lobes exhibited a negative correlation (Figure 3, Table 5).

**Table 5:** Brain regions associated with ALFF values that correlated with MoCA scores in T2DM patients.

Brain regions	BA	x, y, z (MNI)	*R*-value	Voxels
**(I) Positively correlated**				
R cerebellum_6, _Crus1 and vermis	NA	21, −57, −27	0.6337	154
L cerebellum_6, cerebellum_Crus1	NA	−45, −48, −24	0.6269	132
R cerebellum_7b, cerebellum_Crus2	NA	42, −39, −48	0.5858	48
B supplementary motor areas	6	0, 0, 69	0.7167	79
**(II) Negatively correlated**				
L inferior frontal gyrus, temporal pole	38/47	−48, 24, −18	−0.6364	127
B cunei and lingual gyri	18/19	−6, −87, 24	−0.7605	724
R middle occipital, temporal gyri	19/37	51, −72, 6	−0.6093	51
R precuneus	7	18, −63, 39	−0.7035	52

A corrected threshold of *p*<0.05 was applied according to Monte Carlo simulations.

BA, Brodmann's area; MNI, Montreal Neurological Institute; L, left; R, right; NA, not applicable; B, bilateral.

## Discussion

Using the ALFF metric of rs-fMRI, abnormal spontaneous brain activity was detected in various regions of the brain in T2DM patients compared with healthy controls. When this activity was evaluated in relation to clinical diabetic variables, particularly vascular indices and cognitive function, there were three main findings. First, two of the frontal regions with decreased ALFF values were found to correlate with diabetic vascular indices. Second, four of the occipital regions with increased ALFF values (e.g., the bilateral cuneus and precuneus, the right lingual and fusiform gyri, and the right middle occipital gyrus) negatively correlated with the MoCA scores. Third, the negative correlations observed between the extracted ALFF values from the occipital regions and the MoCA scores were verified by voxel-wise correlation analyses between full-brain ALFF values and MoCA scores.

### Neurophysiological tests

Both MoCA and MMSE were used to evaluate global cognitive function for both groups. While there was no difference in the MMSE results for the two groups, the MoCA scores were significantly lower for the T2DM group. This may be due to the fact that the MoCA is a more sensitive screening tool for vascular cognitive impairment than the MMSE [45], [46], and has also been found to be superior in detecting mild cognitive impairment in patients with T2DM [47]. T2DM patients also had a poor performance on the CDT compared to the healthy controls. Conceptually, the CDT is viewed as a visuospatial task and is sensitive to right parietal pathology [48]. However, numerous studies have recently demonstrated that it also reflects frontal-lobe functions, such as executive functioning and attention [49], [50]. Thus, in relation to the present study, poor performance on the MoCA and CDT suggest that the global cognitive decline exhibited by the T2DM patients of this cohort was multi-dimensional and may be due to damage in visuospatial- and executive-related brain regions such as the occipital and frontal regions.

### Decreased ALFF values in the frontal lobe

Two frontal regions exhibited a decrease in ALFF values, the bilateral MPFC and the subcallosal gyrus. In particular, the former was found to correlate with severe microvascular disease (i.e., UACR). This observation is consistent with the results of a previous study where cerebral blood flow in the frontal regions was found to be reduced in T2DM patients compared with the controls [20]. The results of the present study also provide neurophysiological insight into the findings of another study where albuminuria measured by UACR was found to be associated with frontal lobe dysfunction independent of SVD in elderly T2DM patients [15].

Another region that exhibited a decrease in ALFF values was the subcallosal gyrus. The subcallosal gyrus is associated with olfaction, and olfactory dysfunction has been reported for patients with diabetes [51]–[53]. Moreover, it has been hypothesized that this dysfunction is due to diabetic mononeuropathy of the first cranial nerve [52]. In the present study, decreased ALFF values were observed in the subcallosal gyrus of T2DM patients and this provides some evidence to support that a “central neuropathy” condition may also contribute to diabetes-related olfactory dysfunction. Moreover, these decreased ALFF values were found to correlate with severe PAD, which is consistent with the previous finding that PAD negatively affects the olfactory capacity of diabetics [54]. However, since olfactory function tests were not conducted in the present cohort, the decrease in ALFF values observed in the subcallosal gyrus could not be analyzed in relation to olfactory function. Therefore, it will be important for future studies to evaluate olfactory function.

### Decreased ALFF values in the cerebellum

Decreased ALFF values were also found in the bilateral cerebellum region 9 and vermis. It is well-established that the cerebellum regulates a wide-range of cognitive functions in addition to its role in motor coordination [55], [56]. Correspondingly, lesions in the posterior cerebellum lobe have been shown to lead to impairment of executive function, spatial cognition, and linguistic processing, even in the absence of cerebellar motor syndrome [57]. Lesions in the vermis may also lead to affective dysregulation [57]. In the present study, decreased ALFF values in these regions in T2DM patients were observed, along with correlations between these decreased ALFF values and MoCA scores. Taken together, these results suggest that disturbed ALFF values for the cerebellum may play a role in T2DM-related cognitive dysfunction.

### Decreased ALFF values in the thalamus, IPL, and supplementary motor cortex

Decreased ALFF values were also found in the bilateral thalamus, the left IPL, and the left superior frontal gyrus. All of these regions exhibit functional heterogeneity, are involved in cognition control via multiple pathways, and connect with the prefrontal cortex [58]–[61]. Therefore, decreased ALFF values that were observed in these brain regions for the current T2DM cohort may explain the multidimensionality of the cognitive decline manifested by the poor MoCA and CDT scores obtained for these patients. However, none of the ALFF values for these regions were found to correlate with the diabetic variables examined. This may be due to the relatively milder vascular disease conditions exhibited by this cohort (e.g., the incidence for PAD and microalbuminuria was 30.8% and 19.2%, respectively), the relatively shorter T2DM duration (median, 7.0 y), and the improved long-term glycemic control achieved by these T2DM patients (mean, 8.3% of HbA1c).

### Increased ALFF values in the visual network

Increased ALFF values were observed mainly in the visual network, particularly in the bilateral occipital and temporal lobes. These increases may be due to the effects of compensating mechanisms. In addition, increased ALFF values for the visual cortices, such as the bilateral cuneus, the fusiform gyrus, the precuneus, and the right middle occipital and lingual gyri, were found to negatively correlate with MoCA scores in the present study. Taken together, these results may suggest that the stronger the compensation, the greater the cognitive decline.

The results of the present study are partly consistent with two recently published studies of T2DM patients [29], [62]. For example, the brain regions with altered ALFF values in T2DM patients were largely consistent across all three studies (e.g., the occipital lobes, the temporal lobes, and the posterior lobe of the cerebellum). All three studies also identified correlations between the altered ALFF values and cognitive function. In particular, both the current study and the study by Cui et al. [62] found that the ALFF values in the occipital lobe (including the right lingual and fusiform gyri and the left cuneus) correlated with the results of the cognitive tests performed, independent of whether these values increased or decreased compared to the controls. These consistent results suggest that T2DM-related cognitive dysfunction may be partly due to disturbances in the ALFF values of the occipital lobe, the temporal lobe, and the posterior lobe of the cerebellum. However, there are some inconsistencies in the specific ALFF changes that were observed. For example, in the study by Cui et al. [62], a decrease in ALFF values in the occipital lobe were observed, while an increase in ALFF values was observed for the same region in the present study. Similarly, ALFF alterations in the middle temporal gyrus observed in the present study were inconsistent with the ALFF alterations reported by Xia et al [29]. These discrepancies may be due to differences in the cognitive stages of the T2DM patients studied. It has been hypothesized that cognitive dysfunction in T2DM patients may occur during a critical period, perhaps coinciding with the emergence of microvascular complications [1], [3], [63]. Progression of cognitive dysfunction associated with ALFF changes in the occipital lobe may also represent the transition from a compensated stage to a decompensated stage. For the present cohort, the relatively younger age of the patients (54.7±10.4 y) compared with that of the Cui et al. study (58.3±7.3 y), as well as the shorter disease duration (median, 7.0 y vs. 9.3±3.8 y, respectively), may represent a compensated stage of occipital lobe ALFF changes compared with a decompensated stage of occipital lobe ALFF changes, respectively. Another possible reason for these discrepancies may be the number of covariates included in the between-group two-sample t-tests that were performed. For example, the number of covariates investigated by Cui et al. [62] was nine, there were three in the study by Xia et al. [29], and there were four in the present study. These differences may influence the ALFF patterns that were observed. Nevertheless, the relationship between ALFF patterns and the various complications associated with diabetes are very complex. Furthermore, it remains to be determined whether some antidiabetic drugs influence ALFF. Therefore, in-depth longitudinal studies stratified for diabetic complications and antidiabetic drugs are needed to clarify these issues.

### Limitations

As a preliminary study, the current cohort was relatively small. Moreover, since the present study was not a longitudinal survey, we could not conclude that the correlations between altered ALFF values and diabetic vascular disease were the result of diabetic vascular disease. Therefore, it will be important to follow these patients and examine whether the alterations of ALFF patterns are consistent with the progression of diabetic vascular disease. Secondly, in terms of the diabetic microvascular disease index, the UACR measurements were based on a single spot urine sample, and this could misrepresent the extent of microvascular damage. Thirdly, although T2DM patients exhibited poorer CDT performances compare to the controls, a correlation between the CDT scores and ALFF values could not be analyzed due to the skewness of the CDT data. Fourthly, the threshold for ALFF analyses (*p*<0.01 for a one-sample test and *p*<0.05 for two-sample and correlation tests) may not have been sufficiently stringent, thereby increasing the risk for type I errors. Lastly, ALFF changes were found to be widespread throughout the brain, which may indicate that a reorganization of the brain network occurs in T2DM patients. The underlying relationship among these regions, which could not be revealed by ALFF analysis, may also be relevant to the mechanisms mediating T2DM-related cognitive decline. Thus, a combination of ALFF analysis and diffusion tensor imaging may represent a promising approach for future studies.

## Conclusions

In the current study, rs-fMRI was used to obtain ALFF values and to detect brain dysfunction in T2DM patients. The association of these factors with diabetic vascular disease was also examined. As a result, disturbances in the baseline brain activity of T2DM patients were further characterized, leading to the hypothesis that ALFF disturbances in the occipital lobe play an important role in T2DM-related cognitive dysfunction, while ALFF disturbances in the frontal lobe play a key role in the relationship between diabetic vascular disease and T2DM-related abnormalities of baseline brain activity. However, additional comprehensive studies are needed to confirm the present results.
